# Visceral Leishmaniasis in a Twin Pregnancy: A Case Report and Review of the Literature

**DOI:** 10.3390/jcm13082400

**Published:** 2024-04-20

**Authors:** Grigorios Karampas, Sevasti Koulouraki, George L. Daikos, Christina Nanou, Leon Aravantinos, Makarios Eleftheriades, Dimitra Metallinou, Panagiotis Christopoulos

**Affiliations:** 1 Second Department of Obstetrics and Gynaecology, Aretaieio University Hospital, National and Kapodistrian University of Athens, 115 28 Athens, Greece; karampasgr@yahoo.gr (G.K.); sevasti.koulouraki@gmail.com (S.K.); laravant@med.uoa.gr (L.A.); melefth@med.uoa.gr (M.E.); panchrist@med.uoa.gr (P.C.); 2 Mitera Hospital, 6, Erythrou Stavrou Str., 151 23 Athens, Greece; gdaikos@med.uoa.gr; 3 Department of Midwifery, University of West Attica, 122 43 Athens, Greece; nanouxv@uniwa.gr

**Keywords:** leishmaniasis, visceral, kala-azar, twin pregnancy, diagnosis, treatment, congenital leishmanial, perinatal outcome

## Abstract

Visceral leishmaniasis (VL), often referred to as kala-azar, is quite rare in developed countries during pregnancy. Only few studies have evaluated its impact on perinatal outcome. It is caused primarily by *Leishmania donovani* or *Leishmania infantum* and presents with a wide spectrum of clinical manifestations from cutaneous ulcers to multisystem disease. Differential diagnosis is challenging as symptoms and signs are insidious, mimicking other diseases. Misdiagnosis can result in severe adverse perinatal outcomes, even maternal/neonatal death. Early treatment with liposomal amphotericin-B (LAmB) is currently the first choice with adequate effectiveness. We report a rare case of VL in a twin pregnancy with onset at the second trimester, presenting with periodic fever with rigors, right flank pain, and gradual dysregulation of all three cell lines. The positive rK39 enzyme-linked immunosorbent assay test confirmed the diagnosis. Treatment with LAmB resulted in clinical improvement within 48 h and in the delivery of two late-preterm healthy neonates with no symptoms or signs of vertical transmission. The one-year follow-up, of the mother and the neonates, was negative for recurrence. To our knowledge, this is the first reported case of VL in a twin pregnancy, and consequently treatment and perinatal outcome are of great importance.

## 1. Introduction

Leishmaniasis, a disease transmitted by vectors, is caused by more than 20 morphologically indistinguishable species of the obligate intracellular protozoan genus Leishmania, which is transmitted by sand flies of the genus Phlebotomus [1,2]. Leishmaniasis, according to the World Health Organization (WHO) [3], is considered as a “neglected tropical disease” as it is associated mainly with impoverished populations and countries, while only limited resources are invested in research for its diagnosis and management [4].

In the realm of tropical pathologies, leishmaniasis occupies the second position in mortality rankings, attributable to its extensive clinical presentation spectrum, encompassing manifestations from cutaneous lesions to complex multisystemic disease [5]. Depending on the parasite’s reservoir host, it is classified as zoonotic or anthroponotic. Infection from leishmania protozoa may also rarely occur through intravenous drug use and blood transfusion [2,5].

*Leishmania donovani* and *Leishmania infantum* are the primary causes of visceral leishmaniasis (VL). In 2020, approximately 13,000 new cases of visceral leishmaniasis were documented and reported to the World Health Organization [6], a number probably underestimated, especially in Africa. Visceral leishmaniasis caused by *L. infantum* is prevalent in the Middle East, Brazil, and the Mediterranean region, encompassing countries such as Greece, Spain, and France. Transmission of *L. infantum* is considered zoonotic, with canine species acting as the principal reservoir hosts in the urban locales of the aforementioned geographic areas [7].

Τhe estimated annual VL incidence in southern European countries is 437 to 639 new cases [8]. In Greece, *L. infantum* is mainly responsible for VL and is transmitted by the phlebotomine vectors, *Phlebotomus neglectus*, *P. tobbi*, and *P. perfiliewi* [9]. In the Greek region, 1038 domestic and imported cases of leishmaniasis were declared in the years 2004–2021, of which 976 (94%) were cases of VL and 62 (6%) cases of cutaneous leishmaniasis. Visceral leishmaniasis is endemic in Greece, with an average annual incidence of 0.5 cases/100,000 individuals in the population (25–86 domestic cases/year), during the period 2004–2021 [10].

The occurrence of VL during pregnancy is notably uncommon in developed countries, and few studies have evaluated its impact on pregnancy progression and outcome [11,12,13,14,15,16,17]. For residents of developed countries, no specific risk factors are identified, apart from traveling in endemic areas [18] while the prevalence of congenital VL through vertical transmission following maternal disease during pregnancy remains unknown [17,18]. Early diagnosis is currently based on serological tests, and liposomal amphotericin-B (LAmB) is regarded as the preferred treatment option providing adequate perinatal outcome [2,6,9,17].

To our knowledge, this is the first reported case of VL during a twin pregnancy (dichorionic diamniotic twins, DCDA) in Greece [11], in which the mother underwent treatment during the antepartum period, and it resulted in the delivery of two healthy late-preterm neonates.

## 2. Case Report

A 32-year-old second gravida woman of Asian origin, permanent resident of Athens, the capital of Greece, for the last 5 years, presented at the emergency department at 22 weeks of gestation, with a dichorionic diamniotic twin pregnancy (DCDA) and a recent onset of symptoms including anorexia, fatigue, fever with chills, and right flank pain. The patient had an uncomplicated vaginal birth of a healthy full-term female neonate in her first pregnancy, three years before, and had a free medical history.

Two weeks prior to the admission, at 20 weeks of gestation, the woman complained of fatigue and low fever not responding to paracetamol. The complete blood count was normal without leukocytosis (WBC: 9600 K/μλ) while the C-reactive protein (CRP) was elevated (CRP: 21.59 mg/L with a cut-off limit for pregnancy at 15 mg/L). Urinalysis and urine culture, as well as polymerase chain reaction (PCR) test for influenza and severe acute respiratory syndrome coronavirus 2 (SARS-CoV-2) infections, were all negative. All other routine prenatal tests including ultrasound examination of the embryos, control for TORCH infections, and clinical examination were normal. One week prior to the admission (at 21 + 3 weeks), the fetal anatomy scan performed revealed two healthy embryos of 418 g (A: female) and 484 g (B: male) without any indication for congenital disease and normal amniotic fluid index (AFI) in both gestational sacs.

At admission, the physical examination revealed tenderness on the right costovertebral angle and increased temperature at 39.0 °C with stable vitals. Laboratory investigation depicted normal level and type of white blood count (WBC: 7400/µL), hemoglobin (HGB) at 11.4 g/dL, low platelets (PLT) at 116,000/µL, elevated CRP at 4.7 mg/dL (with a cut-off limit for pregnancy at 0.9 mg/dL), and slightly elevated liver enzymes (AST: 36 U/L, ALT: 41 U/L). All other laboratory parameters, including urine tests, were normal. The obstetric ultrasound confirmed a DCDA pregnancy of 22 weeks with normal percentiles of fetal growth, normal AFI, and normal Doppler parameters of umbilical arteries in both embryos. The ultrasound examination of the upper abdomen was also unremarkable, while no hepatosplenomegaly was observed. Based on the above, the initial differential diagnosis of febrile pyelonephritis or acute cholecystitis was excluded.

The woman was hospitalized and received as initial treatment 1.5 g/day of intravenous Cefuroxime Axetil divided in two doses. As the COVID-19 pandemic was ongoing and widespread, new PCR tests for SARS-CoV-2 and influenza virus were performed, excluding once more both pathogens. Thereafter, the pregnant woman gradually developed cyclic incidents of high fever (up to 39.8 °C) with severe rigors every 10–12 h, lasting approximately for 1–2 h with normal temperature between the incidents. Additionally, a mild gradual dysregulation of all three cell lines became apparent on the fourth day of hospitalization, with WBC dropping at 5200/µL presenting normal white blood cell type, anemia (HGB at 10.0 g/dL), and thrombocytopenia (PTL:95,000/µL). In addition, CRP (10.2 mg/dL) and liver transaminases as well as γ-GT and ALP levels gradually rose (AST: 58 U/L, ALT: 58 U/L, ALP: 166 U/L, γ-GT: 48 U/L). Moreover, the auscultation of the lungs and chest X-ray were negative for underlying pathology, and there were no palpable lymph nodes. Finally, urine, vaginal/cervical discharge, and blood cultures were negative for infection.

As the initial antibiotic treatment had no effect on woman’s symptoms and signs, and based on the cycle and regular incidents of high fever with rigors a thorough panel for the detection of infectious diseases including leishmaniasis, salmonella, brucella, toxoplasmosis, rubella, cytomegalovirus, Epstein–Barr virus, herpes simplex virus type I–II, and listeria was ordered on the fourth day. The next day, the positive rK39 enzyme-linked immunosorbent assay (ELISA) test initially confirmed the diagnosis of VL, which was further established by the positive indirect immunofluorescent antibody test (IFAT) for leishmaniasis.

Taking into consideration that the patient was a citizen of Greece with no history of recent travel abroad, it was conjectured that *L. infantum* constituted the most probable subspecies. Intravenous LAmB was administered at a dose of 3 mg/kg/day for seven days, with a total dose of 21 mg/kg. After 48 h, the woman’s clinical condition improved with episodes of fever and rigors becoming milder and totally resolved on the fourth day of treatment. On the last day of the therapeutic regimen, PTL started to rise, and the woman was discharged from the hospital after the last dose.

The follow-up included weekly full blood count, blood biochemical profile, clinical examination, and ultrasound evaluation of the fetuses’ wellbeing, during the first month. One month later, all blood cell lines (WBC: 9500/μL, HGB: 10,8 g/dL, PTL: 221,000/μL) as well as all biochemical parameters, including CRP and liver function, had normalized. Thereafter, the follow-up was continued on a monthly basis according to the national guidelines, and the third trimester obstetrical ultrasound examination at 32 weeks revealed a reassuring biophysical profile for both embryos with normal Doppler parameters. Nevertheless, according to a growth scan, a discrepancy between the two embryos in estimated fetal weight (EFW) started to be apparent with embryo-A at 1402 g (estimated fetal weight percentile: 22.2%) and embryo-B at 1623 g (estimated fetal weight percentile: 57.3%). Two weeks later, the estimated fetal weight percentile was 17% for embryo-A and 42% for embryo-B with normal Doppler parameters in both embryos and AFI for embryo-A at 5.2 cm.

The patient delivered via elective cesarean section two healthy neonates (embryo A: female, 2090 g, embryo B: male, 2740 g) at 35 + 3 weeks of gestation due to fetal growth restriction (FGR) and oligohydramnios of embryo-A according to the last ultrasound examination during pregnancy. Both neonates had normal APGAR scores and were transferred to the neonatal intensive care unit (NICU) for stabilization and further evaluation due to maternal history of leishmaniasis during the antenatal period. Routine blood tests, heart ultrasound examination, transient-evoked otoacoustic emissions (TEOES), ophthalmological examination, and head ultrasound scan were normal in both neonates. The rK39 blood test for neonatal leishmaniasis was negative in both neonates as well as blood tests for cystic fibrosis, TORCH infections, and metabolic disorders. The mother and her infants remained healthy and had a normal 12-month follow-up without any indication of recurrence.

## 3. Discussion

### 3.1. Clinical Presentation

To the best of our knowledge, this represents the first documented case in the scientific literature reporting a twin pregnancy complicated with leishmania, specifically providing clinical manifestations, diagnosis, laboratory data, treatment, and perinatal outcome, including one year follow-up of both neonates.

Leishmania is a parasitic disease that has traditionally been classified by its clinical image in leishmania parasites causing tegumentary leishmaniasis (TL) and those causing VL. Tegumentary leishmaniasis (TL) is characterized by chronic painless ulceration lesions of the epithelial surfaces with an indurated border that gradually heals leaving an atrophic scar, presenting either as localized/diffuse cutaneous leishmaniasis (CL and DCL, respectively) [12,13] or mucocutaneous leishmaniasis (MCL) [14,15,16]. On the contrary, VL is a disease primarily caused by *L. donovani* and *L. infantum* (synonym *L. chagasi*), which cause generally indistinguishable major clinical manifestations [17,18,19].

It is important to differentiate VL from other infectious diseases, such as typhoid fever, brucellosis, histoplasmosis, etc. [17,18] as well as leukemia or lymphoma, in the presence of hematological abnormalities, as there is extensive overlapping among those diseases regarding clinical symptoms and signs [18,19]. Diagnosis might be difficult as the severity of the infection presents a broad range of clinical presentations from asymptomatic infection to VL and a rare form of hemophagocytic lymphohistiocytosis (HLH) [20,21,22,23,24,25,26,27,28,29,30,31,32,33,34].

The absence of symptoms in the asymptomatic infection demonstrates the host system’s capability to manage the parasitic presence, which remains viable lifelong and may reactivate in an immunosuppressed status. Subclinical infections may be detected via leishmania skin testing or interferon-gamma release assays [4,20,21]. On the contrary, in VL (black fever in Hindi), the incubation period varies from few weeks to several months. The onset of symptoms is usually insidious or subacute, like in our case, and mostly includes malaise, fatigue, fever, moderate hepatomegaly, splenomegaly, lymphadenopathy, and gradually darkening discoloration of the skin [2]. Splenomegaly is usually more severe than hepatomegaly, presenting with left upper quadrant discomfort and fullness. Rarely, in the case of parasite invasion in the intestine, diarrhea and malabsorption might occur. Considering the wide range of symptoms and signs, it should be mentioned that, as in our case, periodic high fever in combination with severe rigors ought to heighten suspicion of a parasitic disease and should be carefully validated when presented in cases of fever during pregnancy.

Parasites replicate in the reticuloendothelial system; thus, they mostly accumulate in the bone marrow, spleen, and liver. Consequently, blood dyscrasias are commonly detected. More specifically, normocytic and normochromic anemia is provoked by bone marrow suppression, hypersplenism, and hemolysis, while thrombocytopenia and hepatic dysfunction contribute to hemorrhagic complications [22]. Marked hypergammaglobulinemia results from polyclonal B cell activation. Neutropenia and eosinopenia are also observed, while an elevated value of neutrophils should raise the suspicion of bacterial secondary infection. A number of complications can arise due to anemia, bleeding, malnutrition, and intercurrent infections. Early diagnosis is the best way to avoid severe complications, especially from the hematopoietic system, as in our case in which dysregulation of all three cell lines was mild and the recovery after treatment quick, starting from the first post-treatment week. A high level of liver enzymes and bilirubin is also observed [20,23]. Late in the course of the disease, marked cachexia, hypoalbuminemia, jaundice, and ascites may present due to severe hepatic dysfunction and hypoalbuminemia [20]. Finally, mild renal impairment may occur in *L. infantum* infection. Studies in Brazil and India [24,25] have also reported a renal injury that is reversible with antileishmanial treatment and includes immune-complex interstitial nephritis or less commonly glomerulonephritis, presenting with proteinuria, hematuria, and elevated creatinine and blood urea nitrogen.

If no treatment is administered, VL is lethal [26,27] within 2 years [24] while fatality rates are 10% even after treatment [29,30,31], with the presence of concomitant HIV infection, severe anemia, jaundice, and cachexia being negative prognostic factors with poor prognosis [29,32]. Finally, HLH is a rare complication of VL that is attributed to excess immune activation and presents with fever, hepatosplenomegaly, and pancytopenia [33,34].

No research has been conducted to explore the potential vulnerability of pregnant women to manifest clinical visceral leishmaniasis following infection. Simultaneously, the incidence of congenital VL due to vertical transmission subsequent to maternal illness during pregnancy remains undetermined [17,35]. To date, the literature has only documented a small number of cases involving VL in pregnant women exhibiting a spectrum of symptoms that vary from intermittent fever, popular rash, fatigue, and splenomegaly to respiratory distress and death [36,37]. Placental abnormalities and decreased amniotic fluid volume have also been reported [17,37]. Survival rates among pregnant women treated for VL have been found to vary. Several studies have reported cure rates as high as 100% [38,39], while other studies have reported case fatality rates of 18% [40]. Adverse pregnancy outcomes including high rates of miscarriages and premature birth have been described leading to low-birth-weight neonates [38,39]. Furthermore, pregnant women with VL seem to present more severe anemia and therefore are in need of blood transfusion (OR 9.3; 95%CI 2.5–34.2) in comparison with non-pregnant women with VL, while more antibiotics (OR 6.0; 95%CI 3.4–10.6) seem to be administered to them due to over-diagnosis of other reasons for fever or due to secondary infections [41]. Postpartum hemorrhage has also been shown to be more common, without increasing maternal mortality [42]. A study has also documented cases of surgical evacuation for the removal of retained products of conception; still, this is mostly associated with preterm vaginal delivery [43]. Even fewer congenital VL cases subsequent to VL during pregnancy have been described and are characterized mostly by prematurity, fever, and hepatosplenomegaly in the infected newborn [16,36] while a case report presented a newborn with congenital VL-induced hepatic and neurologic impairment [44].

### 3.2. Pathophysiology of Leishmania Infection during Pregnancy

The pathophysiology of leishmania infection in pregnant women is intricate, given that pregnant individuals represent a unique patient group. This complexity is attributed to the modifications in the maternal immune system throughout pregnancy, which can exhibit both pro-inflammatory and anti-inflammatory characteristics, varying with the gestation period [45]. It is still a point for future research to determine whether there is a relationship between the different trimesters in pregnancy and the susceptibility for developing VL. Additionally, there are no data either for the prevalence of congenital VL or for the risk factors for vertical transmission such as the week of gestation [35,46].

Vertical transmission of leishmania has been observed in veterinary studies; still, the underlying mechanisms cannot be extrapolated to humans due to physiologic differences between animal models and humans [36]. Human vertical transmission is believed to take place via the hematogenous transplacental route [36,46] or through the exchange of blood from mother to child during labor [47]. Transmission after birth, particularly via breastfeeding or through the transfer of deceased parasites, their DNA, or their molecules, is not considered a possibility [47]. The most probable method of transplacental transmission has been confirmed by the identification of parasites in placental, fetal, and newborn tissues in various animal studies. Leishmania parasites have been inspected in the placenta of a stillbirth delivered from a mother with known VL [48]. DNA of a leishmania species was also verified by positive PCR results on the cord blood and the peripheral blood of a premature neonate with intrauterine growth restriction, born from an HIV-positive mother with VL during pregnancy [44]. Moreover, reports have delineated cases of congenital transmission of VL from women exhibiting either asymptomatic or subclinical infection profiles [49].

The clinical manifestations of leishmania infection in pregnancy seem to be the result of interplay between parasite virulence and the host immune response to the parasites’ presence [50]. Despite the known predilection of leishmania species for proliferation within the cells of the mononuclear phagocyte system (MPS), their dissemination cannot be precluded in the context of relative immunotolerance in the gestational environment [51,52]. Thus, the colonization of the placenta by leishmania may alter its structure, enabling parasite transmission to the fetus [48,53,54]. There are different hypotheses about the mechanism of placental invasion and destruction, mostly from studies in the congenital transmission of other microbes such as *T. cruzi* [55,56] and Zika virus [57,58]. Possible mechanisms are the primary targeting of Hoffbauer cells, the placental macrophages [59,60], the parasite-mediated degeneration of the trophoblast [61,62], and villous vessel thrombosis [48,63].

Although congenital transmission is probably underestimated, there are cases of both symptomatic and asymptomatic women delivering in utero-infected neonates [37,54]. Animal studies have corroborated transplacental transmission of the parasite in both VL and TL in laboratory and wild animals [64,65,66,67]. This placental pathology is possibly associated with intrauterine growth restriction, stillbirth, and preterm labor [63,68], while the destruction of the protective maternal–fetal barrier may permit the transmission of the parasite in utero.

Another crucial aspect of leishmania infection is the delicate balance of maternal immunological response between the elimination of the parasite and the protection of the ongoing gestation [69]. Recent findings shed light on the particular roles of distinct T-cell subgroups and cytokines in managing maternal infection and maintaining balance within the gestational environment [70]. T-cell differentiation is determined by variable factors [71], and although the humoral immunologic response is maintained during pregnancy especially at the placenta level, the leishmania species are intracellular microbes that cannot be eliminated by humoral immunity [72]. Therefore, the maternal immune response is challenged by the combative needs of the maternal immunotolerance to the allogeneic fetus versus the necessity to respond to the invading parasite [73,74].

### 3.3. Diagnosis

Epidemiological, clinical, and laboratory evidence is used to diagnose VL. Diagnostic tools to confirm the clinical suspicion of VL include histopathology by visualizing the amastigotes in tissue or blood, molecular methods, and serology testing [2]. The ideal tissue specimen for diagnosis (i.e., histopathology, molecular parasitology methods, or culture) are the bone marrow, the spleen, the secondly enlarged lymph nodes, and the peripheral blood, which should be preferred in immunocompromised individuals [75,76,77,78,79]. Bone marrow and spleen are the most aspirated organs. Bone marrow aspiration presents sensitivity 70% (50–80%) and is usually considered a safe procedure [75]. Spleen aspiration shows a greater sensitivity of 96% (gold-standard approach) but is associated with risk of hemorrhage (1/1000) [76] or bowel perforation and should be performed only by experienced hands and only if platelet count and coagulation tests are at safe levels [42,75,76]. A definite diagnosis requires visualization of amastigotes, usually within the macrophages [42], in tissue smears of the affected organs. Leishmania amastigotes are spherical with a large nucleus, and they involve the presence of kinetoplasts, which are extranuclear DNA and are characteristic for the identification of leishmania among other amastigotes. Upon a negative or inconclusive result, serum antibodies should be looked for [2]. The sensitivity of a culture is generally 60–85%, depending on the parasite load in the sampled material [77,78,79]. Promastigotes may appear four weeks after inoculation, but growth usually occurs within two weeks [77,78].

Nevertheless, molecular tools are gradually replacing microscopy and culture. Molecular techniques are commonly used in developed countries. Among these methods, PCR exhibits high sensitivity levels, which can differ based on the type of tissue sample [80,81,82,83,84,85], achieving as much as 95–97% in spleen aspirates [86]. Polymerase chain reaction is used for the detection and typing of the parasite, while quantitative polymerase chain reaction (qPCR) may be used as a biomarker for response to treatment [87]. A recent study on HIV-negative patients with VL in East Africa found a correlation between the qPCR of the blood of patients two months after the initiation of treatment and tissue parasite load [88]. Polymerase chain reaction is not necessary a confirmatory test in the case of definitive histopathologic diagnosis.

New promising approaches, such as a gene signature in a transcriptomics approach [89,90] and metagenomic next-generation sequencing (mNGS), may be used to complement other routinely used molecular diagnostic tools. Thus, unexpected microorganisms that cannot be detected in culture may be identified, clarifying the differential diagnosis through a simple blood sample. These new tools may also provide more information about the variable species and their antimicrobial susceptibility [91]. There may be limitations to the use of molecular parasitology methods as opposed to biomarkers of cure as they reflect the disappearance of the parasite and not the development of immune response [81,83,92,93].

Serologic tests are also in use for the early differential diagnosis of VL as the infection prompts excessive polyclonal B-cell activation, leading to [94] polyclonal gamma-globulinamia consisting mainly of IgG antileishmanial antibodies, reflecting largely the Th2 immunological response. In areas with advanced laboratories, serology may be used on a negative or inconclusive histopathology, culture, or molecular test with high clinical suspicion, as mentioned above. In endemic regions with limited laboratory settings, serologic testing may be used as a confirmatory test in patients with a clinical diagnosis of leishmania [84,95]. Still there are some drawbacks in serology; in these endemic regions, positive antibodies may be observed in asymptomatic residential individuals with subclinical infections [84,85,96]. Furthermore, serology cannot be used for recovery or relapse follow-up, as it takes months to years for antibodies to return to null values [21,97]; thus, a positive result cannot be considered as definitive proof of active disease. Finally, serology cannot be used in immunocompromised individuals due to high false negative results [42]. For historical reasons, we mention the formol-gel (aldehyde) test that has been used since the 1920s, in which a drop of aldehyde in the serum of the patient could transform it into gel, but only in the advanced stages of the disease [98,99].

The serologic tools currently used, with comparable accuracy, are the Direct Agglutination Test (DAT) and the recombinant kinesin antigen (rK39). The DAT is a useful diagnostic tool in low-income countries as it is read by eye, requiring less equipment [100]. A meta-analysis on the accuracy of DAT revealed that its sensitivity and specificity were 94.8% and 85.9%, respectively [96]. The rK39 is a useful antigen in ELISA assays with variable sensitivity and specificity, depending on the region. In particular, sensitivity is counted up to 100% in the Indian subcontinent [101,102,103] and 61.5–91% in East Africa and Brazil [92,96,104] while specificity is lower at 81.2–96.4% [96]. The rK39-based tests are easily and quickly performed, giving reproducible results, and are therefore suitable for early diagnosis of VL, just like in our case. The rK39 is also the only rapid diagnostic test developed for VL that demands minimal equipment and is easily used in developing countries [42,96,105,106].

Analysis of cytokine responses requires an advanced laboratory and is considered a promising diagnostic tool as a marker of infection and a biomarker monitoring the response to treatment or a possible turn to sepsis [107]. Recently, a monoclonal antibody-based multiplex capture ELISA, utilizing five different monoclonal antibodies against leishmania proteins, demonstrated enhanced sensitivity of ≥ 93% and a specificity of 100% in analyzing urine samples from Brazil and Kenya [108]. Currently, urine antigen tests are rarely used in clinical practice due to their low sensitivity (64%) despite their high specificity (93%) according to a Cochrane meta-analysis [109].

Other assays including the leishmanin skin test (LST) (also called the Montenegro skin test) measures type IV cell-mediated immunity. Intracutaneous injection of inactive leishmania parasites induces induration that is evaluated after 48–72 h [110]. It is a valuable epidemiologic tool as it can detect the population’s immunity by identifying the susceptible individuals when it is negative. It should not be used for a VL diagnosis as it is negative in HIV and in active VL [111], while a positive response usually develops 2 to 24 months after clinical recovery [86,112,113]. Finally, Interferon Gamma Release Assay (IGRA) may be considered as an alternative to LST for identification of patients with asymptomatic infection, and it can be positive in patients with protective immunity or during active VL [93,114,115].

Regarding the diagnosis and follow-up of vertical transmission, there are no established protocols. Vertical transmission in the neonate should be confirmed by a bone marrow aspirate (gold standard) [17] or alternatively by a positive PCR result immediately after delivery. A PCR test later after birth should not be considered diagnostic due to the possibility of vector transmission [54]. Some experts recommend conducting a histopathological examination of the placenta to detect the presence of the parasite [17]. Congenital VL symptoms and signs appear to develop within the first 12 months of life; therefore, asymptomatic offspring should undergo serology testing throughout the first year of life [17]. Nevertheless, the information found in the literature regarding the role of serological tests in monitoring newborns presents conflicting views [54]. In our case, normal blood count and CRP of the neonates in combination with the absence of symptoms/signs of the disease as well as the negative rK39 test excluded the active infection of the two offspring.

### 3.4. Treatment and Follow-Up

Although specialized techniques are required for the species identification, this is not necessary for treatment decisions regarding VL, as they are based on disease severity, geographic origin, and the presence of HIV and/or other coinfections. Agents with efficacy against VL include pentavalent antimonial drugs, miltefosine (the first oral drug for treatment of VL), paromomycin, and amphotericin B (AmB). As long as pentavalent antimony was used as the first-line therapy of VL, the cases of VL during pregnancy used to be treated postpartum, until the development of LAmB, which is the only medicine (category B) approved by the Food and Drug Administration (FDA) for VL during pregnancy [116]. Still, due to the high cost of LAmB, pentavalent antimony is the drug of choice in low-income endemic regions leading to treatment resistance and failures, as well as side effects on the pregnancy, such as abortions and stillbirths [117,118]. Miltefosine is contraindicated during pregnancy because of the risk for stillbirth and teratogenicity observed in animal experimentation [4]. Resistance against antimonials, and potentially miltefosine, is currently increasing, which is a significant disadvantage in the treatment of leishmaniasis [119].

Liposomal amphotericin B has an improved tolerance and safety profile, demonstrating fewer adverse effects to the mother, compared to conventional deoxycholate AmB. Side effects of AmB that have been observed include nephrotoxicity (distal renal tubular acidosis, arginine vasopressin resistance), electrolyte abnormalities (renal potassium sparing), fever with rigors at the beginning of infusion [120], and rarely malaise, rash, and bone marrow suppression. Liposomal amphotericin B consists of amphotericin B packaged with cholesterol and other phospholipids within liposome [121]. This structure prolongs the duration of the total AmB in plasma; stabilizes the drug concentration in blood and tissues; reduces free AmB in the plasma; increases the uptake of liposomes by the tissues, especially at liver and spleen [122]; and minimizes interaction with mammalian cell membranes, thus reducing the drug toxicity [123]. Animal studies have proven that there is no fetal damage from treatment with LAmB during pregnancy [124]. Furthermore, neither maternal death nor pregnancy loss, or vertical transmission of VL, or other adverse effects on the fetus have been observed in various case reports of patients treated during different stages of pregnancy [37,125].

Up to the time of this article was written, there have been no pregnancy-specific universal guidelines, including dosing and standardized dosing times for the use of LAmB [2] in the treatment of singleton or multiple pregnancies, like in our case. During pregnancy, physiologic changes lead to an increase of 50% in glomerular filtration rate and renal blood flow, an increase of 30–40% in circulating plasma volume, and a drop of 20–40% in serum albumin concentration, consequently affecting the medication pharmacokinetics. Amphotericin B crosses the placenta, achieving therapeutic concentrations in the fetal circulation. The ratios of cord blood/maternal blood levels of LAmB have been found to range from 0.38 to 1 [126]. A study concerning obesity showed that the use of total body weight instead of ideal body weight increases the overall concentrations of LAmB and AmB, increasing, as a result, the risk of side effects from LAmB, especially nephrotoxicity [59]. As LAmB is considered a safe medicine for pregnancy, randomized controlled studies for optimal dosing and further research on pregnancy-specific pharmacokinetic properties of LAmB are warranted.

Several studies conducted on the leishmaniasis treatment efficacy of LAmB have demonstrated cure rates ranging from more than 88% in Africa [127,128] to 98–100% in Europe [129,130,131,132]. In 2005, the World Health Organization (WHO) recommended a cumulative dose of 20 mg/kg as sufficient for high cure rates in immunocompetent VL patients worldwide. The FDA recommendation is a LAmB dose of 3 mg/kg on days 1 to 5, 14, and 21 for a total cumulative dose of 21 mg/kg [124]. In our case, the 3 mg/kg/day dose was used for seven consecutive days, a dose that proved to be effective as it resulted in quick clinical improvement and cure of the woman.

Clinical evaluations are generally conducted to determine the effectiveness of treatment, which is characterized by the resolution of fever within one to two weeks and spleen size normalization within a month. In case of immunocompetent patients with clinical response, no additional testing should be performed, and is recommended patients be followed-up clinically for a year as most relapses occur within 6–12 months of the completion of treatment [133,134]. Immunocompromised patients should ideally be followed up lifelong or until efficacious immune reconstitution. In case of equivocal clinical response, Anti-rK39 immunoglobulin IgG1 in either ELISA or rapid test may be used as an effective test of cure, while conventional serologic tests should be avoided as they remain positive for months to years post-treatment [21,42].

A special reference to leishmaniasis-infected HIV-positive pregnant women should be made as their therapeutic management is challenging [46]. The combination of curative and maintenance LAmB dosage along with antiretroviral therapy provides only partial cure and protection, as treatment failure and vertical transmission have been observed [46], and PCR for an indefinite follow-up is recommended [135]. The etiology of the treatment failure may be attributed to the underlying maternal immunosuppression that affects the parasite elimination. The recommended therapeutic scheme of LAmB in this patient group is 4 mg/kg for a cumulative dose of 40 mg/kg, often followed by secondary prophylaxis with 3 to 5 mg/kg LAmB per month [44,135].

### 3.5. Cases and Case Series of Visceral Leishmaniasis in Singleton Pregnancies

Visceral leishmaniasis (VL) exerts an equivalent pathophysiological influence on both singleton and twin gestations. This comprehensive literature review delineates the most important aspects of the disease’s manifestation during pregnancy, encompassing geographical distribution, clinical presentations, diagnostic approaches, therapeutic interventions, and perinatal outcomes. It also incorporates a supplementary file (Table S1) that details exceedingly select elucidative rare cases and case series in singleton pregnancies [9,35,37,38,40,41,44,46,136,137,138,139,140,141,142]. Notably, the incidence of VL in singleton pregnancies within developed countries is markedly low, presenting with a spectrum of clinical signs and symptoms. Such presentations necessitate heightened clinical vigilance to initiate further diagnostic evaluations to either confirm or exclude the presence of this parasitic infection. In the context of singleton pregnancies, LAmB remains the therapeutic modality of choice, yielding optimal perinatal outcomes when its administration is commenced promptly following the onset of infection. Nevertheless, the occurrence of adverse complications, including miscarriage during the first trimester, fetal growth restriction, stillbirth, preterm labor, and maternal death, remains significantly elevated, particularly in cases characterized by delayed diagnosis and therapeutic intervention.

## 4. Conclusions

Visceral leishmaniasis must be considered in the differential diagnosis of unexplained fever with atypical systemic symptoms during pregnancy, particularly when risk factors are present, routine laboratory tests are inconclusive, and there is periodic fever accompanied by rigors [143]. The latter symptoms, in the form of intensive, short, periodic episodes of high fever in combination with severe rigors triggered the suspicion of a parasitic disease in our case and should be carefully evaluated when present during pregnancy. Early diagnosis during pregnancy and subsequent treatment with LAmB has been shown to reduce maternal morbidity, vertical transmission, and obstetrical complications. Still, as VL remains a neglected disease, more research is needed for the ideal diagnostic and therapeutic management in the sensitive period of pregnancy. Liposomal amphotericin B at a dose of 3 mg/kg/day (total 21 mg/kg) seems to be efficient for the treatment of VL during pregnancy not only in singleton but also in the rare case of a twin pregnancy.

## Data Availability

The original contributions presented in the study are included in the article/Supplementary Materials, further inquiries can be directed to the corresponding author/s.

## Supplementary Materials

The following supporting information can be downloaded at: https://www.mdpi.com/article/10.3390/jcm13082400/s1, Table S1: Select case reports and case series of visceral leishmaniasis in singleton pregnancies.
