# Supplementary material for: Visceral Leishmaniasis in a Twin Pregnancy: A Case Report and Review of the Literature
Source: J Clin Med. 2024 Apr 20;13(8):2400. doi: 10.3390/jcm13082400 (PMC11051246; doi:10.3390/jcm13082400)
Supplement: Supplementary file 1 [file jcm-13-02400-s001.zip › jcm-2900564-supplementary.pdf]

Table S1: Select case reports and case series of visceral leishmaniasis in singleton pregnancies.

Authors	Year	Country	n	Symptoms - Signs	Diagnosis	Treatment	Outcome
Pagliano et al. [35]	2005	Italy	5	Fever-hepatosplenomegaly	Antileishmanial antibody titre.<br>Cultures were attempted from bone marrow aspirates.<br>Zymodeme analysis of Leishmania strains obtained.	LAmB<br>3 mg/kg/day on days 1–5 and 10	Anti-Leishmania antibodies became undetectable by month 6. No relapse was observed.<br>Five healthy newborns were delivered; Apgar scores between 7 and 9, and weight between 3200 and 3700 g<br>At delivery, four newborns had detectable levels of anti-Leishmania antibodies; all became negative within 6 months.
Mueller et al. [38]	2006	Soudan	12	Fever of >2 weeks duration and either splenomegaly or at least two of the following: inguinal lymphadenopathy, hepatomegaly, weight loss, recurrent epistaxis or anaemia.	Direct agglutination test (DAT; ≥6400),<br>rapid diagnostic dipstick test (rK39; DiaMed-IT-Leish®)<br>or lymph-node aspiration	LAmB 3–7 mg/kg on days 1, 2, 3, 4, 10, and 15	100% cured when discharged<br>75% still pregnant at discharge<br>No spontaneous abortions during treatment<br>Two healthy infants born during the study<br>One premature infant died after treatment was complete
Figueiró-Filho et al. [46]	2008	Brazil	5	Mean gestational age was 28.7 ± 7.8 weeks.<br>Fever was reported in all cases, three patients reported weight loss, and four reported abdominal pain.	A positive test for anti-leishmania antibody determined by indirect immunofluorescence.<br>Demonstration of amastigote forms of Leishmania in material	LAmB<br>3 mg/kg/day for 20 days	Maternal: Followed up for at least 1 year and considered to be healthy, free of disease, and in adequate nutritional status<br>Neonatal: Follow up for at least 1 year considered to be healthy, free of disease, and in adequate nutritional

					obtained by bone marrow puncture and stained on a slide by the method of Romanovsky (Giemsa, Wright, Leishman or Diff Quick) and/or by in vitro culture in specific medium (NNN), with detection of promastigote forms<br>Bone marrow puncture was performed in the newborns after delivery		status
Sinha et al. [139]	2010	India	3	No available information	Positive rK39 rapid diagnostic test (DiaMed-IT-Leish) or a parasitological diagnostic test result	LAmB at a total dose of 20 mg/kg divided into<br>5 mg/kg on days 0, 1, 4, and 9	Successfully treated. LAmB was considered safe and effective.
Zinchuk et al. [141]	2010	Ukraine	1	Pregnancy of 28-32 weeks.<br>No further available information.	No available information.	LAmB 3 mg/kg on days 1–5 and on day 10.	Periodic rise in temperature without apparent cause during LAmB treatment.<br>Amastigotes of Leishmania infantum identified from bone marrow examination, and patient received further treatment.
Ritmeijer et al. [142]	2011	Ethiopia	2	History of fever for >2 weeks, malaria excluded, in combination with wasting, and either splenomegaly or	Suspected primary VL rK39 rapid diagnostic<br>- if above negative, test DAT with a high titer (≥1:6,400) confirming VL.	LAmB six infusions of<br>5 mg/kg on alternate days	Maternal: Discharged without a test of cure but with good response to treatment.<br>Neonatal: No information provided.

				lymphadenopathy	- intermediate DAT titer (1:800–1:3,200): tissue aspiration (spleen or lymph node) for microscopy and demonstration of leishmania amastigotes to confirm VL.<br>Suspected VL relapse: tissue aspirate microscopy for all patients with a prior history of VL treatment.		
Mescouto-Borges et al. [37]	2013	Brazil	2	Fever >38 °C, weight loss, productive cough, dyspnea, mucocutaneous pallor, oedema of lower limbs	IFAT test in sera samples to detect antibodies against Leishmania.<br>Bone marrow smears were stained with Giemsa<br>PCR on bone marrow samples to detect 120 bp of the conserved region of parasite kDNA	LAmB,<br>3 mg/kg/day for seven days	Preterm emergency caesarean section due to maternal compromise (30^{+6}/40) in case 1 and fetal distress/splenomegaly/hydronephrosis (35^{+4}/40) in case 2<br>Maternal death due to DIC in one of the cases<br>Both neonates symptomatic with positive bone marrow PCR
Salih et al. [137]	2014	Eastern Soudan	23	No available information	Suspected primary VL rK39 rapid diagnostic<br>-if above negative, test DAT with a high titer (≥1:6,400) confirming VL.<br>-intermediate DAT titer (1:800–1:3,200), lymph node aspiration for	LAmB at a total dose of 30 mg/kg, divided into 10 doses of 3 mg/kg on consecutive days.<br>Slow responders received up to a	Treatment responses at 6 month follow-up visits: final cure: 43%, lost to follow-up: 38%, relapse: 7%, death: 6%, unknown: 6%.

					microscopy and demonstration of leishmania amastigotes confirmed VL.<br>Suspected VL relapse: Tissue aspirate microscopy for all patients with a prior history of VL treatment.	total dose up to 50 mg/kg.	
Silva et al. [140]	2015	Brazil	1	Fever, diarrhoea, hepatosplenomegaly	Demonstration of amastigote forms of Leishmania parasite on a bone marrow smear	LAmB<br>4.8 mg/kg/day for 7 days and additional dose on day 10	Patient showed clinical improvement, however, without eradication of the parasite.<br>Transplacental transmission not documented.
Panagopoulos et al. [9]	2017	Greece	1	Symptoms and signs of fatigue and cachexia and hepatosplenomegaly	Demonstration of amastigote forms of Leishmania parasite on a bone marrow smear	LAmB<br>3 mg/kg/day for 5 days followed by two more doses on days 14 and 21	Maternal: normal laboratory findings and liver and spleen sizes one month after treatment. The patient remained healthy at 14 months after integration of treatment with no signs of relapse<br>Neonatal: Delivered 2 months after treatment, with normal weight and Apgar scores. The infant became serologically negative within 6 months of being born. Remained healthy at 12 months after birth.
Adam et al. [40]	2018	Sudan	45	No available information	No available information	No available information	Case–fatality rate of 18%;<br>n=30 (77%) full-term deliveries of a live neonate, n=6 (15%) preterm deliveries, n=2 (5%) spontaneous abortions, and n=1 (2%) stillbirth.

							Recorded causes of death were hepatic complication (n=5; 63%), bleeding manifestation (n=2; 25%), and severe anemia/heart failure (n=1; 13%)
Cunha et al. [138]	2019	Brazil	1	24/40, with a two month history of daily high fever, asthenia and headaches.	Anti-rK39+	LAmB<br>3 mg/kg/day for 7 days	Treatment with LAmB showed significant clinical and laboratorial improvement.<br>The newborn was born healthy at term, with delivery performed without complications.
Pekelharing et al. [41]	2020	South Soudan	85	History of fever more than 2 weeks and splenomegaly and/or lymphadenopathy and/or wasting	Suspected primary VL rK39 rapid diagnostic<br>- if above negative, test DAT with a high titer (≥1:6,400) confirming VL.<br>- intermediate DAT titer (1:800–1:3,200): tissue aspiration (spleen or lymph node) for microscopy and demonstration of leishmania amastigotes to confirm VL.<br>Suspected VL relapse: Tissue aspirate microscopy for all patients with a prior history of VL treatment.	LAmB 5 mg/kg for 6–12 doses (30–60 mg/kg total).	Overall, initial cure rates were 96.5% and the mortality was 1.8%;<br>20% of patients experienced an adverse pregnancy outcome, mostly first trimester miscarriages or third trimester premature deliveries.<br>No information on neonatal outcomes.

Argy et al. [44]	2020	France	1	VL relapse: asthenia, weight loss, and nausea	Microscopy on peripheral blood and bone marrow aspirates confirmed by a PCR assay, which found parasite burdens of 3 × 105 and 4.3 × 107 Leishmania parasites per mL, respectively.	LAmB 4 mg/kg/day for a total dose of 40 mg/kg, followed by LAmB 5 mg/kg every 15 days until delivery	Placenta: few intracellular Leishmania amastigotes found during the microscopic evaluation of the placenta, confirmed by positive PCR. Spontaneous vaginal delivery at 36 weeks. The newborn was severe IUGR <5th percentile, hepatosplenomegaly, and mild axial hypotonia associated with a subfebrile condition, therefore diagnosed with congenital VL and received an additional course of LAmB.
Niamh O'Grady et al. [136]	2023	USA	1	34/40 skin lesions to the nose, forehead, and mucosal thickening and a large nasal soft tissue mass (mucocutaneous leishmaniasis)	PCR on skin biopsy	LAmB 5 mg/kg/day (days 1–7), followed by 4 mg/kg weekly, total LAmB dose of 55 mg/kg over 8 weeks	Spontaneous labor 35/40 of a healthy preterm neonate.

DAT: direct agglutination test; DIC: Disseminated intravascular coagulation; IFAT: immunofluorescence antibody test; IUGR: intrauterine growth restriction; kDNA: kinetoplast DNA; LAmB: liposomal amphotericin-B; PCR: polymerase chain reaction
